# Learning a Markov Logic network for supervised gene regulatory network inference

**DOI:** 10.1186/1471-2105-14-273

**Published:** 2013-09-12

**Authors:** Céline Brouard, Christel Vrain, Julie Dubois, David Castel, Marie-Anne Debily, Florence d’Alché-Buc

**Affiliations:** 1 IBISC EA 4526, Université d’Évry-Val d’Essonne, 23 Boulevard de France, 91037, Évry, France; 2 LIFO, Université d’Orléans, 45067 Orléans, France; 3 CEA/IRCM/LEFG, 91057 Evry, France; 4 Université d’Évry-Val d’Essonne, 91037, Évry, France; 5 INRIA-Saclay, AMIB, LRI umr CNRS 8623, Université Paris Sud, Orsay, France

## Abstract

**Background:**

Gene regulatory network inference remains a challenging problem in systems biology despite the numerous approaches that have been proposed. When substantial knowledge on a gene regulatory network is already available, supervised network inference is appropriate. Such a method builds a binary classifier able to assign a class (Regulation/No regulation) to an ordered pair of genes. Once learnt, the pairwise classifier can be used to predict new regulations. In this work, we explore the framework of Markov Logic Networks (MLN) that combine features of probabilistic graphical models with the expressivity of first-order logic rules.

**Results:**

We propose to learn a Markov Logic network, e.g. a set of weighted rules that conclude on the predicate “regulates”, starting from a known gene regulatory network involved in the switch proliferation/differentiation of keratinocyte cells, a set of experimental transcriptomic data and various descriptions of genes all encoded into first-order logic. As training data are unbalanced, we use asymmetric bagging to learn a set of MLNs. The prediction of a new regulation can then be obtained by averaging predictions of individual MLNs. As a side contribution, we propose three *in silico* tests to assess the performance of any pairwise classifier in various network inference tasks on real datasets. A first test consists of measuring the average performance on balanced edge prediction problem; a second one deals with the ability of the classifier, once enhanced by asymmetric bagging, to update a given network. Finally our main result concerns a third test that measures the ability of the method to predict regulations with a new set of genes. As expected, MLN, when provided with only numerical discretized gene expression data, does not perform as well as a pairwise SVM in terms of AUPR. However, when a more complete description of gene properties is provided by heterogeneous sources, MLN achieves the same performance as a black-box model such as a pairwise SVM while providing relevant insights on the predictions.

**Conclusions:**

The numerical studies show that MLN achieves very good predictive performance while opening the door to some interpretability of the decisions. Besides the ability to suggest new regulations, such an approach allows to cross-validate experimental data with existing knowledge.

## Background

Gene regulatory network inference has received a lot of attention over the last decade due to the abundance of high-throughput data. A gene regulatory network (see for instance [1]) usually refers to a set of genes whose expression varies over time due to the inhibitive or inductive roles of regulators. Deciphering these regulations at work in the cell will provide a thorough understanding of the cell behaviour and will eventually aid in controlling or repairing when needed. Inference of gene regulatory networks as a problem of empirical inference fits the framework of machine learning as described in [2]. Three main families of inference algorithms have been developed so far: (1) unsupervised model-free approaches that use information theory to extract a non-oriented graph of dependence between variables, (2) unsupervised reverse-modeling approaches that model the network behavior as a (dynamical) system [3] and (3) supervised edge prediction approaches that focus on the graph of regulation and only predict the presence/absence of regulations [4-7]. In the first family, relevance networks like ARACNE [8], CLR [9] and TD-ARACNE [10] use a mutual information score between the expression profiles of each pair of genes and given a threshold, decide to predict an interaction or not. The second family is based on model of behavior of the network, either static or dynamic. In case of static models devoted to steady-state data, Gaussian Graphical Models (GGM) [11,12] allow to build a linear regression model that expresses how one gene can be predicted using the set of remaining genes. Interestingly, GGM build a network using partial correlation coefficients, providing a stronger measure of dependence compared to correlation coefficients used in relevance networks. A powerful approach to regression and network inference based on an ensemble of randomized regression trees [13] has also proven to outperform competitors in inferring gene regulatory networks in recent DREAM competitions. Bayesian networks [14] provide another important approach in static modeling. Learning a Bayesian network involves learning the acyclic oriented graph that describes the parental relations between variables and the conditional probabilities that govern the behavior of the network. While appropriate to gene regulation cascades, Bayesian networks cannot, however, model cycles in the network. Other models incorporating dynamical modeling have therefore been proposed in the literature: dynamical Bayesian networks and differential equations [15-17].

Taking a different angle, supervised edge prediction methods build a decision function that associates a class label to a pair of vertices (genes or proteins) without searching for a model of the network behavior. These methods assume that the network to infer is partially known and that information on the vertices are available. They have been mainly developed for protein-protein interaction network inference, using kernel methods [18-23]. The principle underlying [20,21] is to build pairwise Support Vector Machines (SVM) with an appropriate definition of kernels between pairs of proteins from a kernel defined between individual proteins. Pairwise kernels can also be combined into a linear combination (usually an average) to deal with multiple sources of information. In [23], another point of view is taken: local models (still SVMs) are attached to each target protein in order to predict whether a candidate protein interacts with the considered target, and these models are then combined. Recently, the work of [22] has shown that the local model is equivalent to a pairwise SVM considering a local definition of a pairwise kernel.

In the case of gene regulatory network inference, the supervised setting of edge prediction has been explored less. It was first introduced by Qian et al. [4] using gene expression as unique descriptor and further developed by Mordelet et al. with the SIRENE method [5]. Similarly to [23], SIRENE estimates a local model for each transcription factor and then combines all local models together. The method requires a list of known transcription factors that serve as targets. Other advances in supervised edge inference concern with the problem of lack of true negative examples and therefore focus on learning from positive only and unlabeled examples. Some methods develop strategies to select reliable negative examples from the unlabeled set and then solve a classical balanced binary classification problem [24,25]; others adjust the probability of being positive estimated by a classifier trained on positive and unlabeled examples [6,7,26].

Choosing between the three kinds of network inference methods, namely model-free, model-driven and supervised approaches, relies on the goal of the study. Model-free approaches give a good first network approximation when only one kind of data is available. Reverse-modeling delivers a model of the network that can be used to predict its behavior but requires a sufficient amount of observations, if possible acquired with different initial conditions or perturbations. Supervised edge prediction is relevant when a sufficiently large set of regulations is known a priori and various sources of gene annotations are available. It will be especially meaningful when the biologist wants to increase the corpus of existing knowledge.

This paper deals with the latter prediction problem. We assume that a directed graph of regulations is known partially for a target set of genes. For instance, it is the result of the biologists’s experience and careful mining of the literature. Besides the graph structure, we also suppose that a set of various descriptors of genes and their products are available for the target set of genes, such as gene expression data, Gene Ontology (GO) annotation, protein-protein interaction and also genes location on chromosomes. Our goal is to build a decision function that predicts if an ordered pair of regulator and regulee candidates belongs to the class Regulation or No Regulation.

In this work, we address four issues raised by supervised edge prediction and implement the whole approach on a new experimental dataset related to the ID2 genetic regulatory network in human keratinocytes. The first issue concerns the available sources of information about genes and proteins. These sources provide multiple views of the data which are by definition heterogeneous and very often highly structured. The second issue is related to network inference interpretability: many of the proposed methods are black boxes, while biologists are interested in how the predictions have been obtained. The third issue, as raised by many authors, deals with imbalanced data: very few positive examples of “regulation” is available compared to the huge number of negative examples of “no regulation”. Finally, the fourth issue we tackle in this paper, concerns the performance assessment of a supervised edge prediction tool. Although the best performance assessment comes when biologists go back to the experimental laboratory to test prediction of new regulations with additional and independent experiments, there is a lot of room for *in silico* studies to measure the ability of an edge prediction tool to provide evidence for regulations. The first and the second issue call for a common framework of representation for all the views of the data. For that purpose, we use first-order logic to represent both data and background knowledge. In order to benefit from the tools of statistical learning and to avoid some of the weaknesses of pure inductive logic programming raised, for instance, in [27], we choose a Markov Logic network (MLN) [28,29] as the edge predictor. MLN allows to make predictions using a set of weighted first-order logic rules, thus providing interesting insights on decisions. The third issue is systematically solved by using asymmetric bagging [30,31], a well known and generic method that converts a classifier devoted to well-balanced tasks to unbalanced tasks, which was also discussed in [6] among other approaches. It is worth noticing that we do not solve the issue of false negative, e.g. the fact that among the “no regulation” examples, there might be “regulation” examples that have not been validated yet. The reader interested by this issue is invited to study the works of Cerulo et al. [7] and Mordelet & Vert [6]. Finally, as a fourth contribution, we define and perform three typical numerical studies that can be drawn in order to test a machine learning method devoted to edge prediction: one is a basic test with artificially balanced samples in which we just test the ability of the learning method to obtain good performance; the second one consists of building a regulation predictor in a realistic setting from unbalanced datasets using asymmetric bagging and measuring its ability to discover regulations that were not known before; in the third last study, we proceed in the same way but test the ability of the classifier to label correctly pairs of genes with genes from the training network and genes coming from a new candidate set. In order to assess the performance of the MLN-based approach, we define a pairwise Support Vector Machine (SVM) devoted to ordered pairs of genes and use it as a baseline using a straightforward simplification of the tensor product pairwise kernel. Kernel-based methods as well as first-order logic provide a framework to take into account different sources and features of the data: in this study, two simple definitions of pairwise kernels that combine multiple pairwise kernels expressing heterogeneous information are proposed. While the goal of the study is to take advantage of the heterogeneity of features to describe a pair of genes, we also study the behavior of MLN compared to pairwise SVM in the case of single source of quantitative information such as gene expression.

In order to show the interest of solving these four issues, we have applied our approach to the ID2 genetic regulatory network in human keratinocytes and a new dataset of gene expression using RNA interference. The ID2 protein (Inhibitor of Differentiation 2) acts as a negative regulator of basic helix-loop-helix transcription factors. Previous studies have suggested a potential role for ID2 in epidermis homeostasis reflected by the high expression level of *ID2* in proliferating keratinocytes and its down-regulation upon the onset of differentiation [32]. However, the precise implications of ID2 in the process, and in particular its genetic interactions, remain largely unknown. In an attempt to decipher the *ID2* genetic regulation network in human keratinocytes, we conducted a transcriptomic analysis by microarray experiments of HaCaT cells presenting stable overexpression or transient knock-down achieved by RNA interference of *ID2* expression. As a starting point, we retrieved the regulatory networks associated with the differentially expressed genes in cells with high and low level of *ID2* from the Ingenuity Pathway Analysis (IPA) database. We selected a subset of these networks with ontologies of interest for the biologists (cell cycle regulation, cancer, gene expression and signal transduction), merged the corresponding networks and kept only the transcriptional/expression regulations between the genes. The resulting network was finally used to label the couples of genes as a training set.

## Methods

### Learning directed edges from a set of known regulations

Let G be the set of genes of interest. We want to learn a function *h* that takes the descriptors of a gene *G*_1_ and a gene *G*_2_ and predicts if the gene *G*_1_ regulates *G*_2_. Two types of descriptors are considered: descriptors of genes, for instance protein locations within the cell, and relationships between genes reflecting, for instance, if two genes are located on the same chromosome. Let us denote by X the set of descriptors on genes and by R the set of relations. A special descriptor expresses the class: given an ordered pair of two genes *G*_1_ and *G*_2_, it is true when *G*_1_ regulates *G*_2_.

In this work we have chosen to use a first-order logic representation, which allows for an easy representation of several objects (here, genes) and their relationships. Facts representing information about objects and their relations are expressed by atomic expressions, called *atoms*. They are written *P*(*t*_1_,…,*t*_*n*_), where *P* is a *predicate* and *t*_1_,…,*t*_*n*_ are terms; a term being either a variable or a constant. In the remainder strings corresponding to constants will start with upper-case letters and strings corresponding to variables with lower-case letters. An atom is said to be *ground* if all its variables are set to specific values. A ground atom can be true or false, depending of the truth value of the property it expresses. It can therefore be seen as a boolean variable.

Descriptors on genes are thus expressed by expressions of the form *Attr*(*Gname*,*V*), where *Attr* denotes the attribute, *Gname* the name of the gene *G* and *V* the value taken by *G* on the descriptor *Attr*. For instance *ProtLoccell*(*Akt1*,*Cytoplasm*) means that the subcellular localization of *AKT1* product protein is the cytoplasm. For sake of simplicity, we have used the name of the gene to define its product. If a gene codes for several proteins, there is no limitation to denote one gene and all its products by different names. A predicate that relates *CodesFor*(*Gname*,*Pname*) is just needed. Relations between genes are expressed by expressions *Rel*(*G*_1_*name*,*G*_2_*name*) where *Rel* denotes the relation satisfied by genes *G*_1_ and *G*_2_. For instance, *Samechro*(*Cth*,*Id*3) expresses that the genes *CTH* and *ID3* are located on the same chromosome. The property that *G*_1_ regulates *G*_2_ is expressed by the predicate *Regulates*(*G*_1_*name*,*G*_2_*name*). Given two genes *G*_1_ and *G*_2_, we aim to predict whether *Regulates*(*G*_1_*name*,*G*_2_*name*) is true or false. In short, when there is no ambiguity on the genes we write *Y* = 1 when it is true, and *Y* = 0 otherwise. We have chosen the probabilistic framework of supervised classification and we search for a classifier *h* that is based on an estimation of the *a posteriori* probability *P*(*Y* = 1|*G*_1_,*G*_2_). It can be more formally written

$$\begin{array}{l} h_{\theta }(x_{1},x_{2})=\text{sgn}(\hat{P}(Y=1|X_{1}=x_{1},X_{2}=x_{2},\\ \;R_{12}=r_{12},B=b)-\theta ), \end{array}$$

where $X_{i}=x_{i}$ represents the description of *G*_*i*_, $R_{12}=r_{12}$ represents the relations between *G*_1_ and *G*_2_ and B=b represents the background knowledge. *θ* is a threshold, whose value will be discussed in the experiments. As shown by this formalization, the learning framework we consider is beyond the classical framework of machine learning in which data is represented only by attributes; it belongs to the ILP (Inductive Logic Programming) domain, a subfield of machine learning that aims at studying relational learning in first-order formalisms [33].

The model we have chosen is a Markov Logic Network, as introduced in [29]. Such a model is defined as a set of weighted first-order formulas. In this paper, we consider only a subset of first-order logic, composed of rules *A*_1_∧…∧*A*_*n*_ ⇒ *Regulates*(*g*_1_,*g*_2_), where *A*_1_, …, *A*_*n*_ are atoms. Such restrictions correspond to Horn clauses. The left-hand side of the rule (*A*_1_∧…∧*A*_*n*_) is called the body of the rule whereas the right-hand side is called the head of the rule.

### Learning a Markov Logic network

Statistical relational learning (SRL) relates to a subfield of machine learning that combines first-order logic rules with probabilistic graphical frameworks. Among the promising approaches to SRL, Markov Logic Networks (MLNs) introduced by Richardson and Domingos [28,29] are an appealing model. An MLN M is defined by a set of formulas *F* = {*f*_*i*_|*i* = 1,…,*p*} and a weight vector **w** of dimension p, where the clause *f*_*i*_ has an associated weight *w*_*i*_ (negative or positive) that reflects its importance. Therefore, an MLN provides a way of softening first-order logic and encapsulating the weight learning into a probabilistic framework.

A Markov Logic Network together with a finite set of constants C, among which the variables can take their values, defines a Markov Network. This Markov Network can be built by associating a node to each ground atom and by defining a link between two nodes when their corresponding ground atoms occur in the same ground formula. As a consequence, the ground atoms appearing together in a ground clause form a clique in the graph. Let us, for instance, consider the following weighted clause, where *g*1 and *g*2 are two variables representing genes:

(1) $$\begin{array}{l} 0.3\;\;\text{Processbio}(g2,\text{Cell\_proliferation})\\ \wedge \;\text{Processbio}(g1,\text{Negative\_regulation\_of\_cell\_proliferation})\\ \Rightarrow \;\text{Regulates}(g1,g2), \end{array}$$

where the predicate *Processbio*(*Gname*,*Proc*) says that gene *G* is involved in the biological process annotation *Proc* of Gene Ontology [34].

Let us suppose that we have two genes *A* and *B*. The clause (1) leads to four instantiated clauses, corresponding to the instantiations of *g*1 and *g*2 with *A* and *B* : 

1. *Processbio*(*B*,*Cell*_*proliferation*) ∧ *Processbio*(*A*,*Negative*_*regulation*_*of*_*cell*_*proliferation*) ⇒ *Regulates*(*A*,*B*)

2. *Processbio*(*A*,*Cell*_*proliferation*) ∧ *Processbio*(*B*,*Negative*_*regulation*_*of*_*cell*_*proliferation*) ⇒ *Regulates*(*B*,*A*)

3. *Processbio*(*A*,*Cell*_*proliferation*) ∧ *Processbio*(*A*,*Negative*_*regulation*_*of*_*cell*_*proliferation*) ⇒ *Regulates*(*A*,*A*)

4. *Processbio*(*B*,*Cell*_*proliferation*) ∧ *Processbio*(*B*,*Negative*_*regulation*_*of*_*cell*_*proliferation*) ⇒ *Regulates*(*B*,*B*)

**Figure 1:**
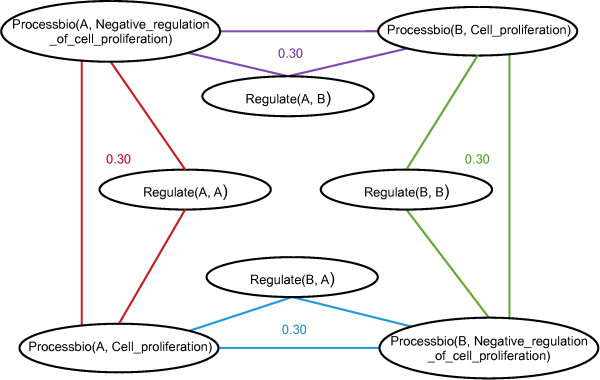
**Schema of a Markov network built from a MLN with** ***C* = {*A,B*}.** The Markov network has been obtained from the clause (1) by considering a set of two constants *A* and *B*. A different color is associated with each instantiated clause.

Variables of the Markov network are the ground atoms occurring in these clauses and they are linked when they occur in the same clause. For instance, the first instantiated clause leads to links between *Processbio(B, Cell_proliferation)* and *Processbio(A,Negative_regulation_of_cell_proliferation)*, *Processbio(B,Cell_proliferation)* and *Regulates(A,B)*, and *Processbio(A,Negative_regulation_of_ cell_proliferation)* and *Regulates(A,B)*. Figure 1 gives the Markov network built from this clause.

A *world* is an assignment of truth values to all possible ground atoms. It is written for short *X* = *x* (*X* denotes the ground atoms and *x* their truth values). The probability of a world *x* is given by:

(2) $$P(X=x)=\frac{1}{Z}\text{exp}\;(\underset{f_{i}\in F}{\sum }w_{i}\times n_{i}(x)),$$

where *n*_*i*_(*x*) is the number of true *groundings* of the clause *f*_*i*_ in the world *x*, and $Z=\sum_{x}P(X=x)$ is the partition function used for normalization.

For instance, if we consider a world where *Processbio(B, Cell_proliferation)*, *Processbio(A,Negative_regulation_ of_cell_proliferation)* are true and the other ground atoms are false, then the first instantiated clause is false in this world, whereas all the other instantiated clauses are true (because their premises are false and the logical implication is false). Thus, the number of true groundings of the clause (1) is 3.

For edge prediction, the aim is to infer a classifier for a specific target predicate, given a set of positive and negative examples and background knowledge. We are thus interested in the Conditional Log Likelihood. Given the predicate *Y* to learn (Y is *Regulates*), we note examples for this predicate *Y*_*j*_ = *y*_*j*_, *j* = 1,…,*n*, and *Y* = *y* if and only if ∀*j*,*Y*_*j*_ = *y*_*j*_. Given evidence *X* which corresponds to descriptors of genes, relations between genes and background knowledge, the Conditional Likelihood (CL) can be expressed using the structure of the Markov network:

(3) $$\begin{array}{ll} \;P(Y=y|X=x) & =\frac{1}{Z_{x}}\exp\;(\underset{f_{i}\in F}{\sum }w_{i}n_{i}(x,y))\\ & =\frac{\exp\;(\sum_{f_{i}\in F}w_{i}n_{i}(x,y))}{\sum_{z}\exp\;(\sum_{f_{i}\in F}w_{i}n_{i}(x,z))}, \end{array}$$

where *n*_*i*_(*x*,*y*) is the number of true groundings of *f*_*i*_ in the world (*x*,*y*).

**Figure 2:**
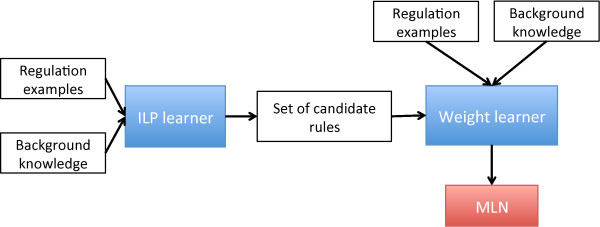
Schema of the two steps of the MLN learning algorithm.

Learning an MLN consists of *structure learning*, i.e., learning the logical formulas, and *parameter learning*, i.e. learning the weight of each formula. Completing these two issues simultaneously raises some complexity issues. Therefore, we have chosen to split the learning task into two subtasks. *Structure learning* can be handled by an inductive logic program (ILP) learner while weight learning can be addressed by maximizing the Conditional Log Likelihood. These subtasks are illustrated in Figure 2.

### Learning the candidate rules with Aleph

The system Aleph, developed by Srinivasan [35], is a well known ILP learner that implements the method proposed in [36]. Aleph, like other relational learners, takes as input ground atoms corresponding to positive and negative examples and background knowledge. It also needs language biases, which restrict the set of clauses that can be generated, thus allowing to reduce the size of the search space. These restrictions can correspond to information specified on the predicates, like the place where they occur in the rule, the types of their arguments or the way they will be used (instantiated or not). In our case, we specified that the predicate *Regulates* occurs in the head of the rule, and the other ones in the body of the rule. Other constraints, such as the maximum number of atoms in a clause or the number of variables, can be defined in order to restrict the form of the rules that can be learned.

The main learning method developed by Aleph, called *induce*, is sketched in the following: 

1. Select a positive example not yet covered by a rule

2. Build the most specific clause *r* that covers this example and that satisfies the language biases from the background knowledge. This clause is called the “bottom clause”.

3. Search a clause more general than the bottom clause: perform a top-down search (from the most general to the most specific clause) in the search space bounded by *r*.

4. Add the clause with the best score to the current theory and prune redundant clauses.

5. Repeat until all positive examples are covered.

### Weight learning

Richardson & Domingos [29] proposed performing generative weight learning for a fixed set of clauses by optimizing the pseudo log-likelihood. Several approaches have been proposed for discriminative learning, where the conditional log-likelihood is optimized instead [37,38]. Huynh & Mooney [39] introduced a weight learning algorithm that targets the case of MLNs containing only non-recursive clauses. In this particular case, each clause contains only one target predicate, thus the grounding of the clauses will contain only one grounded target predicate. This means that the query atoms are all independent given the background atoms. Because of this special assumption on the structure of the model, their approach can perform exact inference when calculating the expected number of true groundings of a clause. Recently, Huynh & Mooney [40] have introduced a discriminative weight learning method based on a max-margin framework.

As we also considered MLNs containing only non-recursive formulas, we used an MAP approach, maximizing the conditional log-likelihood penalized by an *ℓ*_2_ norm constraint:

$$\begin{array}{ll} \;f(w) & =\log P(Y=y|X=x,w)-\lambda \overset{2}{\underset{2}{\left||w|\right|}}\\ & =\overset{n}{\underset{j=1}{\sum }}\log P(Y_{j}=y_{j}|X=x,w)-\lambda {\left||w|\right|}_{2}^{2}, \end{array}$$

with

$$\begin{array}{ll} \;P(Y_{j} & =y_{j}|X=x,w)\\ & =\frac{\text{exp}\;(\sum_{i\in F_{Y_{j}}}\;w_{i}n_{i}(x,y_{\left[Y_{j}=y_{j}\right]})}{\text{exp}\;(\underset{i\in F_{Y_{j}}}{\sum }\;w_{i}n_{i}(x,y_{\left[Y_{j}=0\right]}))\;+\;\text{exp}\;(\;\underset{i\in F_{Y_{j}}}{\sum }\;w_{i}n_{i}(x,y_{\left[Y_{j}=1\right]}))}, \end{array}$$

where $F_{Y_{j}}$ is the set of clauses concluding on the target atom *Y*_*j*_, and $n_{i}(x,y_{\left[Y_{j}=y_{j}\right]})$ is the number of true groundings of the *i*th clause when the atom *Y*_*j*_ is set to the value *Y*_*j*_. For finding the vector of weights **w** optimizing this objective function, we used the limited-memory BFGS algorithm [41] implemented in the software ALCHEMY [42].

### Materials for inference of the ID2 genetic regulatory network

#### Data

We conducted a transcriptomic analysis of microarray experiments of HaCaT cells presenting distinct expression levels of *ID2*. We analyzed three conditions: wild-type cells (wt), stable overexpression (prcID2) or transient knock-down achieved by RNA interference (siID2) of *ID2* expression and their corresponding controls. Differentially expressed genes in prcID2 or siID2 versus the corresponding control cells were identified by a t-test analysis using a p-value cut-off of 0.005, a fold-change threshold of 1.5 and Benjamini & Hochberg multiple testing correction [43]. The resulting genes were mapped to genetic networks as defined by IPA tools and the significantly enriched networks associated with cell cycle regulation, cancer, gene expression and signal transduction were merged. In this merged network, only edges and their associated nodes (genes) corresponding to expression/transcriptional regulations were conserved. Genes with incomplete information for all the features were removed. This process led to the selection of a network containing a set of 63 genes, denoted by $G_{A}$.

In order to use MLNs, we need to describe known properties of genes within the first-order logic setting.

#### Encoding data

Three low level predicates have been defined to reflect the corresponding experimental conditions: 

● The predicate *Expwt*(*Gname*,*L*) states that the expression level of gene *G* in the wild-type cells is *L*. In the following results, expression levels values were discretized using equal width discretization [44]: we divided the interval of gene expression values into 5 intervals of equal width.

● Similarly, the predicate that states that the expression level of gene *G* is *L* is *Expsiid*2(*Gname*,*L*) when the expression of *ID2* has been decreased, and *Expprcid*2(*Gname*,*L*) when it has been increased.

Three other predicates express an increase, a decrease or a lack of change of the expression level between the experience on the wild-type cells and the other experiences: *Expmore*(*Gname*,*Exp*), *Expless*(*Gname*,*Exp*) and *Expsame*(*Gname*,*Exp*), where *Exp* is either *Prcid*2 or *Siid*2.

In order to characterize regulatory interactions, we used other features describing genes. Some of these features concern proteins and not directly genes. 

● **Physical interaction between proteins**: Physical interaction between proteins can provide a hint about the role played by the genes coding for these proteins. In our study, we used the protein interaction data from the IntAct database [45]. We encoded the information of a physical interaction by a predicate containing the name of the genes that are assumed to code the proteins: *Inteprot*(*G*_1_*name*,*G*_2_*name*).

● **Subcellular localization of proteins**:

● Another interesting information about proteins is their localization in the cell. All proteins were analyzed using the Ingenuity Pathway Analysis Knowledge Base (Ingenuity Systems, www.ingenuity.com), and we encoded the information on the subcellular localization by a predicate *ProtLocCell*(*Gname*,*Loc*) where *G* is the name of the gene that codes the protein and *Loc* is the name of the cellular compartment where the protein was found.

● **Biological processes**:

● We used Gene Ontology [34] to describe the genes by the biological processes in which they are involved. To do so, we have defined a predicate *Processbio*(*Gname*,*Proc*), which says that a gene *G* is involved in the process *Proc*.

● **Chromosomal location of genes**:

● We extracted the genes location on chromosomes and chromosomal bands from the Entrez Gene database [46]. This information is encoded by the predicates *Locchro*(*Gname*,*Chro*) and *Locband* (*Gname*,*Arm*_*begin*,*Band*_*begin*,*Arm*_*end*,*Band*_*end*). From these predicates, we built two other predicates that we used instead: *Samechro*(*G*_1_*name*,*G*_2_*name*) and *Sameband*(*G*_1_*name*,*G*_2_*name*). These predicates provide information on the proximity between the gene locations of *G*_1_ and *G*_2_.

### Choice of a baseline for comparison

In the results, we present a comparison with two pairwise Support Vector Machines (SVMs) used as a baseline approach. Contrary to local classifiers, pairwise classifiers do not need an assumption about known transcription factors: any ordered pair of genes can be processed without any prior. As SVM is built from the definition of a similarity between input data, we need to define a kernel between ordered pairs of genes. We say that two ordered pairs of genes (*G*_1_,*G*_2_) and (*G*_3_,*G*_4_) are similar if the regulator candidate *G*_1_ is similar to the regulator candidate *G*_3_ and similarly, the regulee candidate *G*_2_ is similar to the regulee candidate *G*_4_. This definition requires to first choose a kernel between single data noted *k* and then writes as:

(4) $$K((G_{1},G_{2}),(G_{3},G_{4}))=k(G_{1},G_{3})k(G_{2},G_{4}).$$

This pairwise kernel is the asymmetric version of the kernel proposed in [20,22] for pairs of proteins to solve supervised protein-protein interaction network inference tasks. Alternative definitions of pairwise kernel have also been proposed, like the metric learning pairwise kernel [47] and the cartesian kernel [48,49].

For the pairwise kernel defined in (4), when *k* is chosen to be gaussian and *G*_1_,*G*_2_,*G*_3_,*G*_4_ have a feature vector description, *K* is also equivalent to a simple unique gaussian kernel built on the concatenation of feature vectors of each pair’s component such as the one proposed in [4]. In the experimental results we present, we defined six gaussian kernels for each feature described previously: gene expressions, differences of gene expression, protein-protein interactions, subcellular localizations, biological processes and chromosomal locations. However the definition proposed in (4) opens the door to different ways of combining the information. We tested two ways of combining kernels that have been proposed in the pairwise SVM framework (see [20] for instance). The first one consists in deriving for each kernel *k*_*i*_, defined as a kernel between single data, a pairwise kernel *k*_*i*_ and averaging them to build a pairwise kernel noted *K*_*pairwisesum*_:

$$\begin{array}{lll} \;K_{\text{pairwisesum}} & ((G_{1},G_{2}),(G_{3},G_{4}))\; & \;\\ & =\frac{1}{6}\overset{6}{\underset{i=1}{\sum }}K_{i}((G_{1},G_{2}),(G_{3},G_{4})).\; & \; \end{array}$$

The second one consists in first averaging the Gaussian kernels and build as final kernel *K*_*sum*_:

$$K_{\text{sum}}((G_{1},G_{2}),(G_{3},G_{4}))=\bar{k}(G_{1},G_{3})\bar{k}(G_{2},G_{4})),$$

where $\bar{k}(G_{j},G_{k})=\frac{1}{6}\sum_{i=1}^{6}k_{i}(G_{j},G_{k})$.

Let us notice that kernels are appropriate tools to gather heterogeneous sources of information into the same framework and that combining multiple kernels allows active data integration. Once an SVM is built it is hard to open the “black box” and interpret the decision function.

## Results and discussion

### Description of the experimental studies

We conducted three numerical studies of the gene regulatory network associated with *ID2* in human cells, which are summarized in Table 1.

**Table 1:** Summary of the three experimental studies

**Study**	**Positive set**	**Negative set**	**Protocol**
1	$R_{1}^{+}$	*R*1,*i* −,*i* = 1,…,30	10-CV on $R_{1}^{+}\cup R_{1,i}^{-}$
2	$R_{1}^{+}$	*R*1,*i*−,*i* = 1,…,30	AB on $R_{1}^{+}\cup R_{1,i}^{-}$, test on $R_{2}^{+}\setminus R_{1}^{+}$
3	$R_{2}^{+}$	*R*2,*i*−,*i* = 1,…,30	AB on $R_{2}^{+}\cup R_{2,i}^{-}$, test on *R*_3_

We conducted three experimental studies on the gene regulatory network associated with *ID2* in human cells. In the table, 10-CV means cross-validation 10 times and AB means Asymmetric Bagging.

In the first study, we considered the set of 106 regulations provided by Ingenuity in 2007 between the genes in $G_{A}$, denoted by $R_{1}^{+}$. All the unknown regulations ($|\bar{R_{1}^{+}}|=3863$) were considered as negative examples. The goal of this first study was to test a Markov Logic Network on a well-balanced classification task.

For the second study, we considered the set $R_{2}^{+}$ of regulations provided by Ingenuity in 2009 for the same set of genes $G_{A}$. We figured out that 51 new regulations have been discovered by Ingenuity between 2007 and 2009 and we were interested in the prediction task on the updated network. Usual bagging applied to an unbalanced dataset will provide biased classifiers. To build a classifier appropriate for an unbalanced prediction task, we used asymmetric bagging [30,31].

In supervised classification, asymmetric bagging consists of performing random sampling only on the over-represented class, such that the number of examples in the subsample is equal to the number of examples in the under-represented class. This way, each generated predictor was trained on a balanced dataset. Their predictions on the test set were combined to provide a single prediction. Studies described in [30,31] have shown that asymmetric bagging provide better results than normal bagging on unbalanced datasets.

In the last study, we solved a network completion task in real conditions. We selected a new set of genes $G_{B}$ and tried to infer the known regulations between the genes of $G_{B}$ and $G_{A}$. Asymmetric bagging was also applied.

The lists of genes in $G_{A}$ and $G_{B}$ are given in the Additional file 1 and details on Aleph parameters are available in the Additional file 2. Regarding Alchemy, we used the implementation of the discriminative weights learning procedure and tested different values of the regularization parameter *λ*.

#### Evaluation metric

We used area under the ROC (resp. Precision-Recall) curves as evaluation metrics, denoted by AUC-ROC (resp. AUC-PR). These curves were obtained by tuning the threshold *θ* from 0 to 1 in order to predict regulations from posterior probabilities. It is well known that a ROC curve shows the behavior of the True Positive Rate (also called *recall*), $\text{TPR}=\frac{\text{tp}}{p}$, according to the value of the False Positive Rate, $\text{FPR}=\frac{\text{fp}}{n}$, while a PR curve assesses the behavior of the *precision*, $\text{Precision}=\frac{\text{tp}}{\text{tp}+\text{fp}}$, according to the value of the *recall*. A ROC curve expresses the price to be paid in terms of wrongly predicted negative examples when retrieving correctly a number of positive cases. A PR curve, usually plot in information retrieval tasks, puts emphasis on the confidence of positive predictions. We standardized our precision-recall curves similarly to what was proposed in [50].

### Average cross-validation measurements on balanced samples

We first tested the performance of an MLN and compared it to that of a pairwise SVM on a well-balanced classification task. To do that, we subsampled the negative example set and generated subsamples of negative examples of the same size as the positive examples set.

The dataset contains a set $R_{1}^{+}$ of 106 positive examples of regulations between the genes of $G_{A}$. We randomly sampled 30 sets of negative examples *R*1,*i*−, *i* = 1,…,30 with $R_{1,i}^{-}\subseteq \bar{R_{1}^{+}}$, and $\left|R_{1,i}^{-}|=|R_{1}^{+}\right|$. Then for each sample we performed a 10-fold cross-validation experiment (10-CV) on $R_{1}^{+}\cup R_{1,i}^{-}$. In each experiment, we first used Aleph, the ILP tool previously described, as a structure learner on the training set. With Alchemy [42], we learned the weights of the MLN defined by the structure obtained with Aleph and then we performed inference on the test set. For the SVM we used Lib-SVM that we fed with the right definition of the two pairwise kernels computed in Matlab. The bandwidth parameter for each of the six Gaussian kernels was chosen to maximize the entropy of the kernel values. Table 2 shows the averaged AUC-ROC and AUC-PR values obtained within a large range of values of the hyperparameter *λ* of the MLN while Table 3 contains the same results for bagged SVM for different values of *C*. As *λ*, *C* is a regularization parameter. It controls the importance of the *ℓ*_1_ norm of the slack variables in the dual expression of the loss function. The results of the MLN are not significantly different from those of the best SVM, the “sum” one. These results are very good both in terms of AUC-ROC and AUC-PR. It is also important to notice that neither the MLN nor the SVM are very sensitive to the value of the hyperparameter. However we have noted that *λ*, the *ℓ*_2_ norm constraint parameter has to be chosen high (larger than 20 to get interesting results).

**Table 2:** Averaged AUCs for cross-validation measurements on balanced samples using MLNs

	**MLN**	
***λ***	**AUC-ROC**	**AUC-PR**
20	80.8 ± 6.1	82.7 ± 5.4
50	84.3 ± 3.5	85.5 ± 4.0
100	**84.4** **±** **2.8**	**86.2** **±** **3.2**
500	83.4 ± 2.7	86.0 ± 2.7
750	83.3 ± 2.8	85.8 ± 2.8

The table reports the averaged AUC values and standard deviations obtained with MLNs for thirty ten folds cross-validation experiments conducted on a regulatory network between the genes in $G_{A}$. The results are reported for different values of the regularization parameter *λ*.

**Table 3:** Averaged AUCs for cross-validation measurements on balanced samples using SVMs

**Pairwise SVM**				
**C**	**Pairwise sum**		**Sum**	
	**AUC-ROC**	**AUC-PR**	**AUC-ROC**	**AUC-PR**
0.001	70.9 ± 3.5	73.1 ± 3.4	82.5 ± 2.3	84.3 ± 2.1
0.01	70.9 ± 3.5	73.1 ± 3.4	82.5 ± 2.3	84.3 ± 2.1
0.1	70.9 ± 3.5	73.1 ± 3.4	82.5 ± 2.3	84.3 ± 2.1
1	76.4 ± 3.1	78.7 ± 3.0	**85.2** **±** **2.8**	**87.3** **±** **2.5**
10	77.5 ± 3.2	79.4 ± 3.5	84.3 ± 3.4	86.3 ± 3.1
100	77.5 ± 3.2	79.4 ± 3.5	84.3 ± 3.4	86.3 ± 3.1
1000	77.5 ± 3.2	79.4 ± 3.5	84.3 ± 3.4	86.3 ± 3.1

The table reports the averaged AUC values and standard deviations obtained with SVMs for thirty ten folds cross-validation experiments conducted on a regulatory network between the genes in $G_{A}$. The column “Sum” shows the results when the pairwise kernel is derived from the sum of genomic kernels, while the column “Pairwise sum” shows the results obtained using the sum of pairwise kernels derived from each genomic kernel. The results are reported for different values of the regularization parameter *C*.

### Prediction on the updated graph

In this second study, we addressed a network completion task while keeping the same set of nodes. Two years after the dataset described previously was obtained, the tool Ingenuity was used again to provide an updated set $R_{2}^{+}$ of regulations between the 63 genes of interest on this date. We noticed that 51 new regulations were discovered by Ingenuity between these two dates. We were therefore interested in the prediction task of the updated graph, i.e. to see if we could retrieve these new regulations from the data of 2007. We used the dataset $R_{1}^{+}$ from 2007 containing 106 regulations as positive training set and tried to infer the 51 new regulations in $R_{2}^{+}\setminus R_{1}^{+}$ using asymmetric bagging. To that end, we randomly sampled 30 negative examples training sets $R_{1,i}^{-}$, *i* = 1,…,30 with $R_{1,i}^{-}\subseteq \bar{R_{1}^{+}}\setminus R_{2}^{+}$ and $\left|R_{1,i}^{-}|=|R_{1}^{+}\right|$.

As negative examples correspond here to absences of regulation, the test examples were all positive in this study. We could therefore compute the proportion of regulations which were correctly predicted as positive ones by the classifier with a threshold selected using a validation set in the following way: for each sampling of the negative examples, 2/3 of $R_{1}^{+}$ and $R_{1,i}^{-}$ were considered for the training set and the remaining regulations were considered for the validation set. We computed each time the F_1_-measure obtained on the validation set for different threshold values between 0 and 1:

() $$\begin{array}{c} F_{1}=2.\frac{\text{Precision}*\text{Recall}}{\text{Precision}+\text{Recall}}. \end{array}$$

We selected the threshold maximizing the averaged F_1_-measure, that is the value maximizing precision and recall at the same time.

Then, for each sampling, we applied the predictor learned on the training set to the 51 new regulations. We averaged the predictions obtained and used the selected threshold value to compute the true positive rate (TPR). The TPR values obtained using bagged MLN and bagged pairwise SVMs respectively are given in Table 4. Again results using the MLN are very good, showing that it is possible to predict new regulations from an existing corpus at a given time. The performance of pairwise SVMs are inverted, the “pairwise sum” SVM achieving the same results as the MLN.

**Table 4:** Prediction of regulations on the updated network

**Bagged MLNs**		
***λ***		**TPR**
50		64.7
100		72.6
500		80.4
750		84.3
1000		**90.2**
2000		88.2
5000		84.3
**Bagged pairwise SVMs**		
**C**	**Pairwise sum**	**Sum**
	**TPR**	**TPR**
0.001	**90.2**	58.8
0.01	88.3	58.8
0.1	88.3	58.8
1	74.5	52.9
10	64.7	43.1
100	64.7	43.1
1000	64.7	43.1

This table lists the true positive rates (TPR) obtained for the prediction of regulations in $R_{\text{2}}^{\text{+}}\setminus R_{\text{1}}^{\text{+}}$ from *R*_1_ using asymmetric bagging with 30 samples for bagged MLNs and bagged pairwise SVMs. The TPR values were obtained using a threshold maximizing the averaged F1-measure on a validation set. Notations are given in Tables 2 and 3.

### Prediction with a new set of genes

For the third statistical analysis, we addressed a network completion task when new candidate nodes are added. We used a dataset refined in the biology laboratory. 209 high confidence differentially expressed genes in prcID2 versus the corresponding control cells were identified. From these genes, we selected 37 genes that were not part of $G_{A}$ and for which we had an annotation for each predicate. These genes were also chosen from the ones that had at least one regulation link with one of the genes from $G_{A}$ or with one gene of this new set. From these 37 genes, we selected a subset of 24 genes, called $G_{B}$, that had at least a biological process annotation from GO in common with genes from $G_{A}$. The goal of this study was to try to complete a known network using an additional set of candidates genes, which is usually the *problem of interest* for the biologists. We used Ingenuity to retrieve the known regulations between genes from $G_{A}$ and $G_{B}$, being aware that when no regulation is mentioned in the literature, it does not mean that it does not exist but only that it has not been discovered yet. We called this set $R_{3}^{+}$.

We used the set $R_{2}^{+}$ of 157 regulations from the dataset $G_{A}$ as the positive examples training set and used asymmetric bagging with 30 samples on *R*_2_. For each sampling we applied the predictor on the sets $R_{3}^{+}$ and $\bar{R_{3}^{+}}$, using descriptors on both set of genes $G_{A}$ and $G_{B}$. We obtained score predictions for each interaction between one gene of $G_{A}$ and one gene of $G_{B}$. Table 5 reports the AUC values computed for bagged MLNs and bagged SVMs. The ROC and PR curves obtained for the hyper-parameters associated with the best values of AUC-ROC are represented in the Additional file 3.

**Table 5:** **Prediction of regulations between the set of genes**$G_{A}$** and**$$G_{B}$$

**Bagged MLNs**				
***λ***	**AUC-ROC**			**AUC-PR**
50		72.8		6.7
100		73.1		7.7
500		73.2		9.2
750		**73.4**		9.5
1000		73.1		9.5
5000		73.0		**9.8**
10000		72.8		9.5
**Bagged pairwise SVMs**				
**C**	**Pairwise sum**		**Sum**	
	**AUC-ROC**	**AUC-PR**	**AUC-ROC**	**AUC-PR**
0.001	62.8	4.0	66.2	7.8
0.01	62.8	4.0	66.2	7.8
0.1	62.8	4.0	66.2	7.8
1	65.3	7.7	67.4	**8.6**
10	65.4	6.1	**67.5**	8.3
100	65.4	6.1	67.5	8.3
1000	65.4	6.1	67.5	8.3

This table lists the AUC-ROC and AUC-PR values obtained for the prediction of regulations between$G_{A}$ and $G_{B}$ for bagged MLNs and bagged pairwise SVMs, with notations given in Tables 2 and 3. These results were obtained using asymmetric bagging with 30 samples on the set *R*_2_.

Although each predictor was trained on a balanced dataset, with same numbers of positive and negative examples of regulation, this test was made under real conditions: we considered the whole set of positive ($|R_{3}^{+}|=55$) and negative examples ($|\bar{R_{3}^{+}}|=2969$) to assess the performance in prediction. On the test-training interactions, the predictor with bagged MLNs performed quite well, showing an AUC-ROC of about 0.73. This was really a very good result which implies low degradation in performance especially for the false positive rate that only slightly increases. The AUC values obtained with bagged MLNs are above the values obtained with the two bagged SVMs. We performed a statistical test in order to compare the AUC-ROC values obtained with the different classifiers. We used the non-parametric test on Mann Whitney statistics developed by [51] and the implementation provided by the R package pROC [52]. The obtained p-values are given in the Additional file 4. We observe from this results that the p-values are less than 0.05 and therefore that the AUC-ROC values of bagged MLNs and bagged pairwise sum SVMs are significantly different. Regarding the comparison between bagged MLNs and bagged sum SVMs, the difference between AUC-ROC values is not significant, indicating similar predictive performance.

AUC-PR of bagged MLNs outperforms the best pairwise SVM. Therefore in a real prediction task, e.g. a network completion task, MLN exhibits a very interesting behaviour, even if the AUC-PR still needs to be increased.

In Table 6, we also present the results obtained for this task when using only gene expression levels as input descriptors. First we notice that, compared to the results in Table 5, the performance of both approaches, SVMs and MLNs, diminish, showing that the additional descriptors play an important role. Second, in terms of AUC-ROC, the performance of bagged pairwise SVMs and bagged MLNs are similar. The p-values obtained by performing a statistical test for the comparison of these AUC-ROC values are greater than 0.3 for all hyper-parameter values. We can therefore deduce that the difference of AUC-ROC between bagged MLNs and bagged SVMs in terms of AUC-ROC is not significant. Third, for all the approaches, AUC-PR values are very low, but bagged SVMs now outperform bagged MLNs. As expected, SVMs are more suitable for dealing with numerical data. We therefore recommend to use the MLN method when different sources of gene descriptions such as symbolic and discrete descriptions are available.

**Table 6:** **Prediction of regulations between the set of genes**$G_{A}$** and**$G_{B}$** when using only gene expression data as descriptors**

**Bagged MLNs**				
***λ***	**AUC-ROC**			**AUC-PR**
50		61.5		2.4
100		62.5		2.5
500		59.5		2.3
750		64.6		2.5
1000		**64.9**		**2.5**
5000		64.0		2.5
10000		62.7		2.4
**Bagged pairwise SVMs**				
**C**	**Pairwise sum**		**Sum**	
	**AUC-ROC**	**AUC-PR**	**AUC-ROC**	**AUC-PR**
0.001	60.2	3.0	62.8	3.9
0.01	60.2	3.0	62.8	3.9
0.1	60.2	3.0	62.8	3.9
1	62.8	4.2	**64.8**	**6.4**
10	60.9	4.8	64.0	6.1
100	60.9	4.8	64.0	6.1
1000	60.9	4.8	64.0	6.1

This table presents the results obtained with bagged MLNs and bagged pairwise SVMs on the third task when using only gene expression data as gene descriptors.

To conclude, we have shown in this section that bagged sum SVM performs well in Task1 and Task3, while bagged pairwise sum SVM performs well in Task2. Contrary to the SVM classifiers, MLNs behaved well in the three tasks. Now another interesting criterion to choose a method for network inference is to measure its ability to provide insights on the taken decisions.

### Resulting logical rules

In addition to the capacity of the built classifier to suggest new regulations, MLNs present the advantage of providing a set of weighted rules that the biologist can check. In general, Aleph learned between 30 and 50 rules for each run, these rules being composed of up to five predicates. This comes from the choice of the parameters of Aleph as described in the Additional file 2. The analysis of the rules that have obtained a high weight shows that some of them exhibit relevant patterns. Among the rules inferred by Aleph, here is an example of four rules which were associated to a high weight in the numerical tests: 

1. *ProtLoccell*(*g*_2_,*Plasma*_*membrane*) ∧ *Expsiid*2(*g*_2_,*Level*3) ∧ *Expsiid*2(*g*_1_,*Level*3) ⇒ *Regulates*(*g*_1_,*g*_2_)

2. *Processbio*(*g*_2_,*Cell*_*proliferation*) ∧ *Processbio*(*g*_1_,*Negative*_*regulation*_*of*_*cel*_*proliferation*) ⇒ *Regulates*(*g*_1_,*g*_2_)

3. *Expsiid*2(*g*_1_,*Level*3) ∧ *Expprcid*2(*g*_1_,*Level*4) ∧ *Expsiid*2(*g*_2_,*Level*4) ∧ *Expprcid*2(*g*_2_,*Level*5) ⇒ *Regulates*(*g*_1_,*g*_2_)

4. *Expprcid*2(*g*_1_,*Level*5) ∧ *Expwt*(*g*_2_,*Level*2) ∧ *Expprcid*2(*g*_2_,*Level*4) ⇒ *Regulates*(*g*_1_,*g*_2_)

The first rule means that a gene overexpressed in transient knock-down of *ID2* regulates overexpressed genes in the same condition and that code for proteins in plasma membrane. Obviously, this rule alone is far too general but within a set of rules with positive and negative weights, it brings a piece of evidence for regulation. The second rule may seem trivial but it has been retrieved from data: it says that genes involved in negative regulation of cell proliferation regulate genes involved in cell proliferation. The next rule means that an increase of the expressions of *G*_1_ and *G*_2_ in the condition of over expression of *ID2* compared to transient knock-down of *ID2* indicates a regulation between *G*_1_ and *G*_2_. Regarding the last rule, it indicates that a high expression value of *G*_1_ in the prcID2 condition and the increase of the expression of *G*_2_ between wild-type condition and prcID2 implies the existence of a regulation between these two genes.

These rules are examples of what has been obtained in a first attempt to build a whole strategy to get a supervised edge predictor. However the quality of the learnt rules strongly depends on the nature of the chosen predicates and the ILP learning phase. We notice that a substantial improvement can be reached in terms of rules if the biologist makes explicit some constraints on the rules. For instance, one might want rules that include at least relations on both input genes in their premises. We will favor this research direction in the future.

Another information that can be extracted from the learnt MLN concerns the statistics of presence of some of the predicates in the premises of the rules. In our experimental studies, chromosomal location of genes did not appear as an important property to conclude about regulation.

## Conclusions

Recent years have witnessed the preeminence of numerical and statistical machine learning tools in computational biology. Among them, kernel-based methods present numerous advantages, including the ability to deal with heterogeneous sources of information by encoding them into similarities (kernels). On top of that, multiple kernel learning allows to select sources of informations thought the learning of sparse linear combination of kernels [19,53,54]. However kernel-based methods remain black boxes: using non linear kernels, the decision function learnt with a SVM is not at all interpretable. This is an inherent drawback of SVMs because biologists are generally not only interested in the prediction made by a classifier but also in the reason why such an example has been labeled in a given way.

This work explores another direction through a new hybrid tool based on first-order logic representation and probabilistic graphical modeling. Once learnt, a MLN provides a weighted set of rules that conclude on the target predicate, here the *regulates* predicate. To our knowledge this work is the first application of MLN to gene regulatory network inference and one of the very first real applications of MLN on noisy and medium scale biological data. As described in the previous sections, learning a MLN involves several steps including data encoding, choice of constraints and hyper-parameters in the ILP learner and the weight learner as well as an appropriate learning protocol scheme for achieving the learning task. All these steps require a high level of collaboration between biologists and computer scientists which is facilitated by the common language of first-order logic. Therefore, in one hand, the encoding process can be seen as a limitation since each new application requires specific work about the choice and the definition of the predicates to be used. Compared to the kernel design, this step is expensive. However, on the other hand, it produces a corpus of interpretable facts and rules encoding the nature of the relationship between genes that the biologist can inspect. Moreover, it is worth pointing out the fact that it is relatively easy in this context to impose known rules or to perform incremental learning at the level of the rule learner. There is also a lot of relevant information that can be made available that we did not incorporate to describe genes. For instance, adding knowledge of regulatory motifs of genes and DNA-binding sites of regulatory proteins, could improve the performance of the predictor. This means that a proper representation of sequences should be described either directly in first-order logic as it was done in [55], or using an extension of first-order logic to sequence variables like those of [56]. This is certainly a direction to be explored in future works.

Another issue is scalability to larger networks composed of thousands of genes. This would be a concern for pairwise kernel-based methods for instance for the later task to compute the Gram matrix between training and test data. For MLN, scaling to a larger number of genes like thousands of genes should be made possible using the latest improvement in MLN learning implemented in FELIX [57] using dual composition.

Another interesting question is to compare decision trees with MLNs. Decision trees are usually built from attribute-value representations but have been extended to first-order logic in [58]. They also provide a set of interpretable rules but in a less general form than in MLNs. In a decision tree, rules are factorisable and a given example to be classified will only satisfy one rule. On the contrary, a MLN devoted to supervised classification a given example can satisfy many rules. Interestingly, combining decision trees to learn compact representations of MLNs has been recently proposed in [59].

Finally the biologist interested in the ID2 genetic regulatory network in human keratinocytes gets two main results from this work additionally to a set of facts and rules describing the network. First, learning such a supervised pairwise classifier can be seen as a cross-validation of both experiments and existing literature. The ability of the learning algorithm to build a good edge prediction tool shows indeed that text-mining and careful curation can produce networks that are consistent. Inversely, the experimental data measured in the wet laboratory are proven to make sense. Second, the last *in silico* study can provide a list of predicted regulations with new candidate genes, some of them being known but some of the others, considered currently as false positive, may involve new regulators and new targets. This calls for an experimental wet lab validation to test the relevance of the potential new regulations.

## Competing interests

The authors declare that they have no competing interests.

## Authors’ contributions

CB checked the encoding of the data in first-order logic, built the new datasets, developed codes for the whole pipeline and tested all the methods. FAB and CV conceived the methodological part of the project, MAD conceived its biological part about the ID2 network. DC and MAD did the experiments in the wet laboratory and provide a preliminary analysis of the data. JD encoded the data and the background knowledge into first-order logic. CV supervised the use of ILP for rule learning and FAB supervised the MLN approach, the asymmetric bagging part and designed with CB the baseline SVM. MAD participated to design the performance assessment with CB and FAB. CB and FAB drafted the document with the help of CV and MAD. All authors read and approved the final manuscript.

## Supplementary Material

Additional file 1 List of gene symbols used in the study. Click here for file

Additional file 2 Choice of Aleph parameters. Click here for file

Additional file 3 **ROC and PR curves obtained for the prediction of regulations between sets**$G_{\text{A}}$** and **$G_{\text{B}}$**.** The figure represents the ROC and PR curves obtained with bagged MLNs, bagged SVMs (sum) and bagged SVMs (pairwise sum) using the hyperparameters associated with the best AUC-ROC values. Click here for file

Additional file 4 P-values of the non-parametric test based on Mann Whitney statistics to compare AUC-ROC obtained with bagged MLNs and bagged pairwise SVMs. Click here for file
