# Supplementary material for: Learning a Markov Logic network for supervised gene regulatory network inference
Source: BMC Bioinformatics. 2013 Sep 12;14:273. doi: 10.1186/1471-2105-14-273 (PMC3849013; doi:10.1186/1471-2105-14-273)
Supplement: Additional file 1 — List of gene symbols used in the study. [file 1471-2105-14-273-S1.pdf]

Dataset

Genes in set $\mathcal{G}_A$: list of symbols

ADRB2, ADM, ASF1B, AKT1, BARD1, BAX, BCL3, CXCL5, CEBPB, CITED2, CDKN2A, CDKN1A, CCNB1, CTH, CHST6, DDIT3, E2F2, EGFR, FEN1, FOXM1, GHR, GDF8, GADD45B, HBP1, HSPA1A, HMGA2, ITGA2, ID3, ID2, IL1A, IL1B, IER3, INHBA, KLF6, LCN2, MATN2, MMP1, ODC1, PHLDA1, PCNA, PSMA3, PPP1CC, PLK1, PAPPA, PLCG1, PLAU, RAB6A, SLC3A2, SMTN, SLC1A3, SERPINE2, SPARC, TFDP1, TGIF1, TCF3, TK1, TMEM97, TIMP1, UPP1, VEGFA, VPREB1, VRK1, YAP1

Genes in set $\mathcal{G}_B$: list of symbols

AHNAK, AURKA, CASP1, CCNA1, CDKN2B, CDKN3, DLG7, E2F1, EPAS1, HSPA5, ITPR3, JAG1, MDK, MMP10, MMP13, MYBL2, NR2F2, PSMB8, RARRES3, RFC4, TNC, TNFSF10, TPT1, TYMS
