# Supplementary material for: Learning a Markov Logic network for supervised gene regulatory network inference
Source: BMC Bioinformatics. 2013 Sep 12;14:273. doi: 10.1186/1471-2105-14-273 (PMC3849013; doi:10.1186/1471-2105-14-273)
Supplement: Additional file 2 — Choice of Aleph parameters. [file 1471-2105-14-273-S2.pdf]

Aleph parameters

The number of new variables that can be introduced in the body of the rule was set to 2, and the maximum number of atoms in a clause was set to 5. Building a rule that rejects all the negative examples is often too constrained in real-life applications. In the same way building a set of rules that cover all the positive examples may lead to build very specific rules. The minimum number of positive examples that a clause must cover was set to 2, whereas the maximum number of negative examples that a clause is allowed to cover was set to 15. We used the m-estimate evaluation function, which is a Bayesian estimation of the accuracy of a clause; the value of m is automatically set to be the maximum likelihood estimate of the best value of m.

Several variants of the learning method used by Aleph to score a clause have been defined. *Induce_cover* is a variant of *induce*, the method used by default, that does not remove positive examples covered by a clause when scoring a new clause. *Induce_max* is another variant that performs the loop on all positive examples and not only on the positive examples not yet covered. We chose to use the last strategy as it returns a set of rules, which does not depend on the order of the positive examples.
