# Supplementary figures and images for: Learning a Markov Logic network for supervised gene regulatory network inference
Source: BMC Bioinformatics. 2013 Sep 12;14:273. doi: 10.1186/1471-2105-14-273 (PMC3849013; doi:10.1186/1471-2105-14-273)

ROC curve

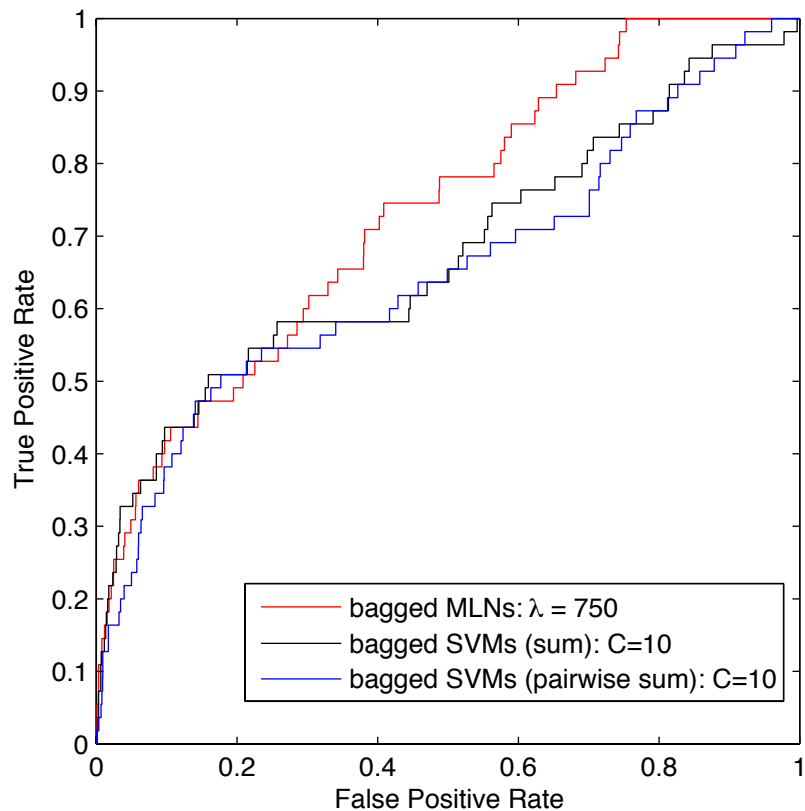

PR curve

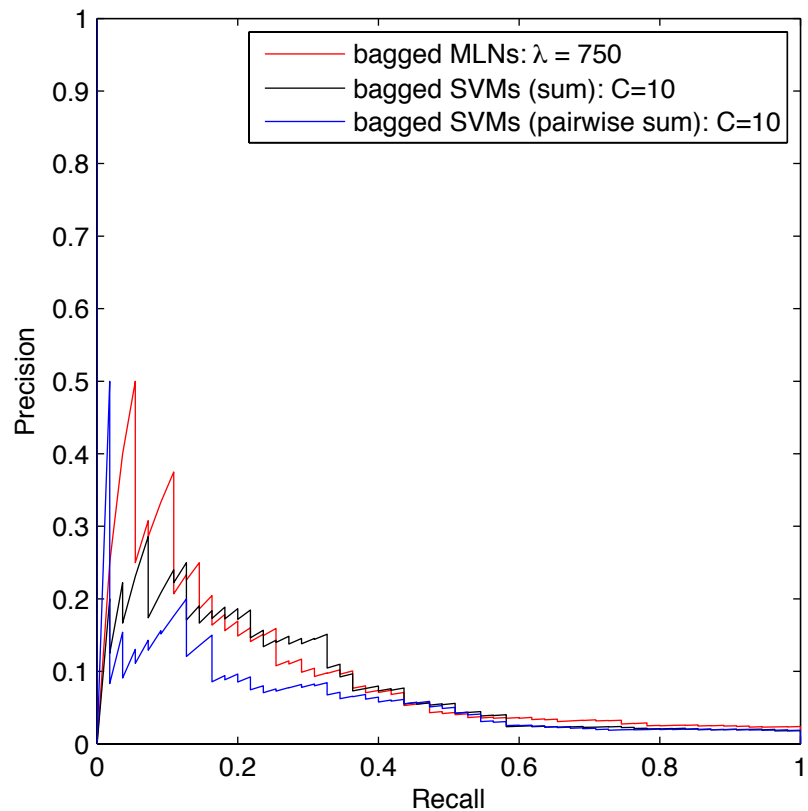

Supplement: Additional file 3 — ROC and PR curves obtained for the prediction of regulations between setsGA and GB. The figure represents the ROC and PR curves obtained with bagged MLNs, bagged SVMs (sum) and bagged SVMs (pairwise sum) using the hyperparameters associated with the best AUC-ROC values. [file 1471-2105-14-273-S3.pdf]
