# Supplementary material for: Learning a Markov Logic network for supervised gene regulatory network inference
Source: BMC Bioinformatics. 2013 Sep 12;14:273. doi: 10.1186/1471-2105-14-273 (PMC3849013; doi:10.1186/1471-2105-14-273)
Supplement: Additional file 4 — P-values of the non-parametric test based on Mann Whitney statistics to compare AUC-ROC obtained with bagged MLNs and bagged pairwise SVMs. [file 1471-2105-14-273-S4.pdf]

	$C = 0.001$	$C = 0.01$	$C = 0.1$	$C = 1$	$C = 10$	$C = 100$	$C = 1000$
$\lambda = 50$	**0.009**	**0.009**	**0.009**	0.064	0.074	0.074	0.074
$\lambda = 100$	**0.007**	**0.007**	**0.007**	0.057	0.065	0.065	0.065
$\lambda = 500$	**0.006**	**0.006**	**0.006**	0.052	0.054	0.054	0.054
$\lambda = 750$	**0.005**	**0.005**	**0.005**	**0.045**	**0.046**	**0.046**	**0.046**
$\lambda = 1000$	**0.006**	**0.006**	**0.006**	0.055	0.056	0.056	0.056
$\lambda = 5000$	**0.006**	**0.006**	**0.006**	0.056	0.056	0.056	0.056
$\lambda = 10000$	**0.007**	**0.007**	**0.007**	0.061	0.062	0.062	0.062

Table 1: P-values of the non-parametric test based on Mann Whitney statistics to compare AUC-ROC obtained with bagged MLNs and bagged pairwise SVMs (pairwise sum). These values are given for a range of hyperparameter C for SVM and λ for MLN.

	$C = 0.001$	$C = 0.01$	$C = 0.1$	$C = 1$	$C = 10$	$C = 100$	$C = 1000$
$\lambda = 50$	0.092	0.092	0.092	0.206	0.213	0.213	0.213
$\lambda = 100$	0.074	0.074	0.074	0.179	0.183	0.183	0.183
$\lambda = 500$	0.060	0.060	0.060	0.158	0.156	0.156	0.156
$\lambda = 750$	0.052	0.052	0.052	0.141	0.138	0.138	0.138
$\lambda = 1000$	0.065	0.065	0.065	0.167	0.164	0.164	0.164
$\lambda = 5000$	0.065	0.065	0.065	0.169	0.165	0.165	0.165
$\lambda = 10000$	0.069	0.069	0.069	0.180	0.176	0.176	0.176

Table 2: P-values of the non-parametric test based on Mann Whitney statistics to compare AUC-ROC obtained with bagged MLNs and bagged pairwise SVMs (sum). These values are given for a range of hyperparameter C for SVM and λ for MLN.
